# Translation, cross-cultural adaptation and validity study of the Menstrual Practice Needs Scale (MPNS-36)

**DOI:** 10.1590/0034-7167-2024-0606

**Published:** 2025-12-08

**Authors:** Maria Eduarda Pascoaloto da Silva, Evelly Vitória Azevedo de Souza, Bianca Dargam Gomes Vieira, Mariana Lourenço Haddad, Ana Paula de Assis Sales, Sonia Silva Marcon, Catchia Hermes-Uliana, Mara Cristina Ribeiro Furlan

**Affiliations:** I Universidade Estadual de Maringá. Maringá, Paraná, Brazil; II Universidade Federal da Grande Dourados. Dourados, Mato Grosso do Sul, Brazil; III Universidade Federal Fluminense. Niterói, Rio de Janeiro, Brazil; IV Universidade Estadual de Londrina. Londrina, Paraná, Brazil; V Universidade Federal de Mato Grosso do Sul. Três Lagoas, Mato Grosso do Sul, Brazil

**Keywords:** Menstruation, Validation Study, Menstrual Hygiene Products, Translating, Women’s Health., Menstruación, Estudio de Validación, Productos para la Higiene Menstrual, Traducción, Salud de la Mujer.

## Abstract

**Objectives:**

to translate, cross-culturally adapt, and validate the Menstrual Practice Needs Scale for the Brazilian context.

**Methods:**

a methodological study, following the stages: initial translation; synthesis; back-translation; synthesis; review by a committee of experts; pre-test; and submission and approval by the author. The committee was composed of 11 judges. Pre-test collection was carried out from July to October 2023 in a city in Mato Grosso do Sul, with 360 high school students. The analysis used the Content Validity Index and Cronbach’s alpha.

**Results:**

the committee achieved a Content Validity Index of 96.7%, but adjustments suggested by judges were incorporated without further analysis. The version was approved for pre-testing, presenting a Cronbach’s alpha of 0.78, with no doubts or suggestions for modification.

**Conclusions:**

the cross-cultural adaptation and validity of the scale for Brazilian Portuguese were considered adequate for application in the target population.

## RESUMEN

**Objetivos:**

traducir, adaptar transculturalmente y validar la *Menstrual Practice Needs Scale* para el contexto brasileño.

**Métodos:**

estudio metodológico, siguiendo los pasos: traducción inicial; síntesis; retrotraducción; síntesis; revisión por un comité de expertos; prueba previa; y presentación y aprobación por el autor. El comité estuvo compuesto por 11 jueces. La recopilación de datos para la prueba previa se realizó de julio a octubre de 2023 en una ciudad de Mato Grosso do Sul, con 360 estudiantes de secundaria. El análisis utilizó el Índice de Validez de Contenido y el alfa de Cronbach.

**Resultados:**

el comité obtuvo un Índice de Validez de Contenido del 96,7%, pero los ajustes sugeridos por los jueces se incorporaron sin mayor análisis. La versión fue aprobada para la prueba previa, presentando un alfa de Cronbach de 0,78, sin dudas ni sugerencias de modificación.

**Conclusiones:**

la adaptación transcultural y la validación de la escala para portugués brasileño se consideraron adecuadas para su aplicación en la población objetivo.

## INTRODUCTION

Menstrual intimate hygiene is crucial to women’s physical, psychological and spiritual well-being and is widely recognized as a fundamental right. Its proper management requires the availability of appropriate hygienic environments and methods as well as adequate materials and information^(1,2)^. The situation known as “period poverty” or “period precarity” refers to the economic and social vulnerability that affects billions of menstruating people globally, due to the lack of access to basic sanitation, toilets and essential items such as sanitary pads^(3)^.

In Brazil, more than 713,000 girls live without bathrooms at home, and around 200,000 female students face precarious conditions when managing menstruation at school, which can cause emotional distress and negatively impact women’s development^(4)^. In response to this reality, Law 14,214, enacted in 2021 and regulated by Decree 11,432 in March 2023, instituted the free distribution of sanitary pads and care related to menstrual health^(5)^. However, the implementation of these policies is still insufficient, as sanitary pads represent only one of the many resources needed to ensure menstrual dignity^(2,6)^.

This scenario is directly related to the lack of studies that adequately reflect the situation of menstrual poverty in Brazil and that enable the development of public policies that are appropriate to the national reality. The “Menstrual Poverty in Brazil” report, prepared by the United Nations Children’s Fund and the United Nations Population Fund in 2021, highlighted the difficulties faced in analyzing the country’s reality in relation to menstrual needs^(4)^. One of the main objectives of this report was to highlight the urgency of conducting more quantitative research and comprehensive data collection to fill this gap and provide insight into menstrual poverty in Brazil.

After reviewing the literature, we identified a knowledge gap: the lack of valid instruments in Brazilian Portuguese to measure the menstrual hygiene needs of Brazilian girls and women. This absence makes correlating national data with results from different cultures difficult. However, the search for suitable instruments revealed the Menstrual Practice Needs Scale (MPNS-36) in the international literature. Developed and validated in Soroti, Uganda, MPNS-36 provides a solid basis for assessing menstrual needs and can be adapted to the Brazilian context^(7)^.

Assessing the factors that influence menstrual hygiene habits and practices requires the use of valid instruments to ensure accurate analyses. Data collection with validated instruments in Portuguese guides nursing practice, contributing to the development of health technologies and improving the quality of care provided^(8,9)^. Thus, the scarcity of translations and cross-cultural adaptations of measuring instruments in this area highlights the need for this research.

## OBJECTIVES

To translate, cross-culturally adapt, and validate MPNS-36 for the Brazilian context.

## METHODS

### Ethical aspects

To conduct this study, authorization was obtained from the corresponding author of the scale to proceed with translation, cultural adaptation and validity. The study was submitted to and approved by the *Universidade Federal de Mato Grosso do Sul* Research Ethics Committee, under Opinion 5.652.278, complying with all ethical and legal requirements for research involving human beings.

### Study design, period and place

This is a methodological study with a quantitative approach, whose methodological framework was proposed by Beaton *et al*.^(10)^. To carry out the process, the following stages were followed: 1) initial translation; 2) translation synthesis; 3) back-translation; 4) back-translation synthesis; 5) review by a committee of experts (judges); 6) pre-test completion; 7) submission and approval by the author. Data collection for the pre-test stage took place from July to October 2023 in two state high schools in a municipality in the eastern mesoregion of Mato Grosso do Sul. This study was conducted according to the COnsensus-based Standards for the selection of health Measurement INstruments guidelines to ensure methodological quality in the translation, cultural adaptation, and assessment of the instrument’s psychometric properties.

### Population or sample; inclusion and exclusion criteria

The study population consisted of four translators, two responsible for the first stage of translation and two for back-translation and 11 assessment judges. While there is no consensus in the literature regarding the exact number of judges that should be included on the committee of experts, it is essential that the members be experts in the field of study and fluent in the original language of the instrument^(7)^. The committee intentionally selected judges through non-probabilistic convenience sampling, taking into account the relevance of professionals’ qualifications and specialization in the subject. The committee included a translator from each phase of the translation process, as well as a linguistics professional, a methodologist, and health and nursing experts^(8,11)^.

The pre-test sample included 360 participants, distributed between two state high schools: 86 female students from school A (35 in the 1^st^ year, 24 in the 2^nd^ year and 27 in the 3^rd^ year) and 274 female students from school B (88 in the 1^st^ year, 88 in the 2^nd^ year and 98 in the 3^rd^ year). It was decided to define the pre-test sample using a proportion of 10:1, as recommended by the original scale study^(7)^. Female adolescents enrolled in high school at the selected schools who had already gone through menarche were included. Those whose menarche had occurred less than six months previously or who had cognitive difficulties that could compromise their understanding and completion of the instrument were not included.

### Data collection instrument

MPNS-36 was developed and validated in Soroti, Uganda^(7)^. It can be self-administered or through an interview. The scale uses a three-point Likert format, with responses such as “never” (0), “sometimes” (1), “often” (2), and “always” (3). MPNS-36 consists of six dimensions, or subscales, that group items together to capture specific information about menstrual practices. Among these subscales, four are applicable to all interviewees, while two are relevant only to those who wash or reuse their menstrual material^(7)^.

The subscales cover topics such as material and home environment needs, transport and school environment needs, material and reliability concerns, change and disposal insecurity, reuse needs and reuse insecurity^(7)^. The scale score is calculated by averaging the responses to the relevant items. Response options have specific values, with “never” being equivalent to 0, “sometimes” to 1, “often” to 2, and “always” to 3. Higher scores indicate a more positive menstrual experience, while a score of 3 suggests that the respondent has no unmet needs and has a well-met menstrual reality^(7)^.

### Study protocol

The first stage was translation into Brazilian Portuguese by two bilingual translators (T1 and T2). Only translators who had Brazilian Portuguese as their native language and were proficient in English were included. T1 was a Brazilian nursing professional with master’s and doctoral degrees in women’s health. They were proficient in English and had experience with translation and cultural adaptation processes. T2 was a sworn translator of Brazilian nationality with a degree in languages and linguistics (Portuguese/English). They were bilingual in Portuguese and English and had experience translating, versioning, and reviewing scientific articles, books, and general documents. These choices aimed to minimize the risk of biases related to language, psychology, culture, theory, and practice. The researcher and translators performed analyses of discrepancies between T1’s and T2’s versions. After the analysis, a synthesis of the two versions was created and called T1-2. The goal of this comparison was to integrate the two translations and resolve any discrepancies, resulting in a single version.

Subsequently, a back-translation (BT) of version T1-2 was performed. Two bilingual translators (BT1 and BT2) who were native speakers of English and proficient in Brazilian Portuguese performed the back-translation (Brazilian Portuguese → English). Both back-translators were native speakers of Brazilian Portuguese and English, and had Brazilian and American nationalities. During the synthesis stage, the translators and the researchers compared the instruments to identify similarities and ensure consistency between the versions. If discrepancies were found, the translators and researchers reached a consensus to correct them, ensuring that the translated version reflected the same content as the original. After the analysis, they created a synthesis of these versions, called BT1-2.

The third stage consisted of assessing the semantic, idiomatic, cultural and conceptual equivalences of MPNS-36. This assessment was carried out by a committee of experts in two phases: the first involved reviewing all the material produced through an electronic form; the second was a meeting between the researchers to resolve discrepancies and reach consensus on the final version.

The committee of experts was composed of 11 healthcare professionals and/or nursing professors, with at least three years of experience in health, public health or women’s health, as well as experience with translation and cultural adaptation, and a linguist. Experience with translation and cultural adaptation was defined based on experts’ previous participation in methodological studies on the topic and/or related publications, as long as they had prior contact with this type of process, without requiring a minimum time or specific number of productions. Professional selection was made through the *Lattes* Platform and nominations^(12)^.

Judges assessed the instrument using a Likert scale from 1 to 4, focusing on four types of equivalence: semantic (preservation of the meaning of words); idiomatic (adequacy of expressions and colloquialisms to the target language); cultural (adaptation of terms and situations to the cultural context); and conceptual (correct representation of specific cultural concepts)^(8)^.

The assessment form was made available on Google Forms^®^, organized into chapters and subdivided into paragraphs to facilitate analysis. Experts had 30 days to complete the assessment and were asked to provide comments whenever the score assigned was less than 4. The analysis of items followed a scale of four levels of agreement: 1 = not equivalent or unclear; 2 = requires major revision or is unclear; 3 = equivalent, requires minor changes or is quite clear; 4 = absolutely equivalent or very clear^(8,11)^. The form included a space for specific suggestions. After the assessments were collected, the suggestions were analyzed, and only items with at least 80% agreement were accepted. Items that did not reach this threshold were reassessed by the committee of experts until the required level of agreement was reached.

The final stage of the study involved administering the pre-final version of the scale to 360 high school students from two selected schools. The study team was trained to ensure proper use of the instrument. Application of the scale was authorized by the ethics committee and the State Department of Education of Mato Grosso do Sul after agreements were made with the principals and coordinators and discussions were held with the teachers of the involved classes. Students were informed about the objective, development, risks, and benefits of the research. Meetings were scheduled to provide additional information and answer questions. During these meetings, assent and consent forms were made available, and parents or guardians were given a minimum of one week to sign and deliver the documents to the research team. Data collection began only after these stages were completed.

**Figure 1:**
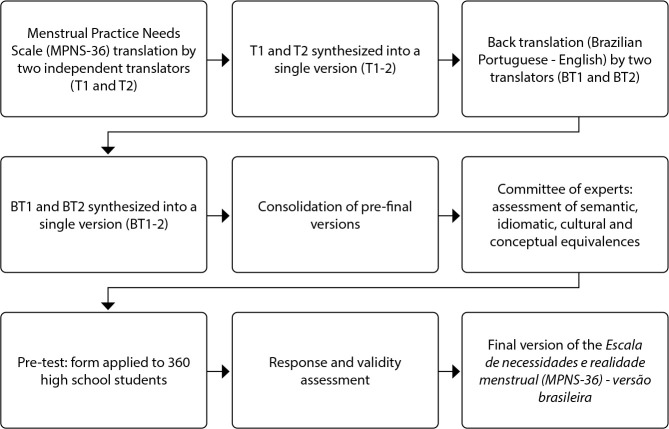
Process of translation, cultural adaptation and validity of the Escala de necessidades e realidade menstrual (MPNS-36) - versão brasileira

Data were collected using participants’ tablets and cell phones connected to the internet via a QR code provided by administrators. The form, made available online on Google Forms^®^, presented the original scale and included a space for suggestions or questions after each question. At the end, the administrators confirmed completion and informed participants that the results would be sent by email, encouraging them to contact the team for further clarification. Data collection took place from July to October 2023, and all items on the form were mandatory, except for comments and questions about reusing the materials. Figure 1 describes the procedures for translation, cultural adaptation, and validity.

*T1 - translation 1; T2 - translation 2; BT1 - back-translation 1; BT2 - back-translation 2.*

### Analysis of results and statistics

The data were organized in a Microsoft Excel^®^ spreadsheet and exported to the Statistical Package for the Social Sciences version 25.0.0.0 for statistical analysis. Content validity was assessed using the Content Validity Index (CVI), which is calculated by the proportion of items that received a score of “3” or “4” on the clarity scale. Items with a score below “4” were revised based on the judges’ suggestions. An acceptable agreement index was established at a minimum of 0.80^(8,10,11)^. We analyzed the reliability and internal consistency of the scale items for the pre-test population responses using Cronbach’s alpha, which ranges from 0 to 1. Values close to 1 indicate greater internal consistency^(13)^.

## RESULTS

The MPNS-36 was adapted and validated for Brazilian culture, receiving the title of “*Escala de Necessidades e Realidade Menstrual (MPNS-36) - Versão Brasileira*”. The Brazilian version is structured in six dimensions or subscales: *material e ambiente doméstico* (material and home environment needs)*; transporte e ambiente escolar* (transport and school environment needs); *preocupações com a confiabilidade do material* (material and reliability concerns); *insegurança com mudanças e descarte* (change and disposal insecurity); *necessidades de reutilização do material* (reuse needs); and *insegurança na reutilização do material* (reuse insecurity).

In the first stage, the translations performed by T1 and T2 were synthesized in version T12. Adjustments were necessary to resolve semantic discrepancies related to the terms “*itens de higiene menstrual*” (menstrual hygiene items), “*período*” (period) and “*ciclo menstrual*” (menstrual cycle), aligning them with the original scale’s language, giving rise to T1-2. Back-translation generated BT1 and BT2. Both back-translations showed high similarity with the original version, but small discrepancies were identified. The final version was chosen based on clarity and the absence of abbreviations; for example, where BT1 used “e.g.”, which is an abbreviation for “*por exemplo*”, the BT2 version was chosen, which used “for example”. Questions that better suited clearer verb tenses and language were used, thus arriving at a synthesis version of the back-translations (BT1-2).

A committee of 11 experts conducted the MPNS-36 semantic, idiomatic, cultural, and conceptual equivalence assessment. Seven of them (63.6%) were nurses, and four (36.4%) were language and linguistics professionals. All of the experts were proficient in English. Three of the experts (27.3%) had experience in translation and cultural adaptation studies, and four (36.4%) were nurses who specialized in women’s health. In terms of qualifications, one had a bachelor’s degree, three were specialists, three had master’s degrees, and four had doctoral degrees. They were between 28 and 50 years old.

The assessment resulted in an overall CVI of 96.7%. The specific CVIs were semantic (96.5%), idiomatic (94.9%), cultural (96.7%) and conceptual (98.5%). Despite the rates above 80%, expert judges made important considerations, leading to significant adjustments in the translated and adapted version for Brazilian Portuguese, but a new analysis was not necessary. Judges’ observations included grammatical corrections, substitutions of colloquial terms and regional suggestions. The title was one of the most modified areas, with two suggestions that improved the understanding of what the scale intends to assess, resulting in the replacement of “*prática*” (practice) with “*realidade*” (reality) of menstruation.

Furthermore, the “*itens de higiene menstrual*” (menstrual hygiene items) term was inadequate in the Brazilian context, which led to the inclusion of a detailed explanation within the scale, mentioning examples such as sanitary pads and menstrual cups, thus becoming: *absorvente/coletor menstrual/tampão/pano de absorção menstrual e/ou outros* (absorbent pad/menstrual cup/tampon/menstrual absorption cloth and/or others). Question 21 replaced the word “*machucasse*” (harm) by “*prejudicasse*” (jeopardize) to avoid ambiguities. It was also decided to modify “basin” to “*pia/bacia/tanque*” (sink/basin/tank), as suggested by experts. The terminology “menstrual cycle” and the response options “never”, “sometimes”, “frequently” and “always” were accepted without changes. Grammatical and agreement corrections were made to improve the scale’s clarity. Following a review, the face validity of the adapted version was confirmed, yielding a total CVI of 97% and indicating a high level of agreement among judges^(4,11)^. Thus, consensus was reached on the final Brazilian Portuguese translation of MPNS-36 (Chart 1).

**Chart 1:** Final translated version of *Escala de Necessidades e Realidade Menstrual (MPNS-36) - Versão Brasileira* approved by the committee of experts

*Escala de Necessidades e Realidade Menstrual MPNS-36 - Versão Brasileira*				
*Opção de resposta*	*Nunca* 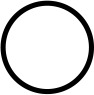	*Às vezes* 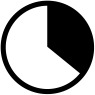	*Frequentemente* 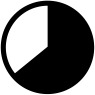	*Sempre* 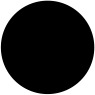
***Durante minha última menstruação...***				
***1-*** *Meus itens de higiene menstrual (absorvente/coletor menstrual/tampão/pano de absorção menstrual e/ou outros) foram confortáveis*				
***2-*** *Eu tive itens de higiene menstrual (absorvente/coletor menstrual/tampão/pano de absorção menstrual e/ou outros) suficientes para trocar com a frequência que quis*				
***3-*** *Eu fiquei satisfeita com a limpeza dos meus itens de higiene menstrual (absorvente/coletor menstrual/tampão/pano de absorção menstrual e/ou outros)*				
***4-*** *Eu pude obter mais unidades do meu item de higiene menstrual (absorvente/coletor menstrual/tampão/pano de absorção menstrual e/ou outros) quando precisei*				
***5-*** *Me preocupei que meus itens de higiene menstrual (absorvente/coletor menstrual/tampão/pano de absorção menstrual e/ou outros) permitissem que o sangue atravessasse para a parte externa das minhas roupas*				
***6-*** *Me preocupei que meus itens de higiene menstrual (absorvente/coletor menstrual/tampão/pano de absorção menstrual e/ou outros) saíssem do lugar enquanto eu os utilizava*				
***7-*** *Me preocupei em como obter mais itens de higiene menstrual (absorvente/coletor menstrual/tampão/pano de absorção menstrual e/ou outros) se eles acabassem*				
***8-*** *Me senti confortável ao levar meus itens de higiene menstrual (absorvente/coletor menstrual/tampão/pano de absorção menstrual e/ou outros) extras comigo enquanto estava fora de casa*				
***9-*** *Me senti confortável ao levar meus itens de higiene menstrual (absorvente/coletor menstrual/tampão/pano de absorção menstrual e/ou outros) extras comigo até o lugar onde os trocava*				
***10-*** *Me senti confortável armazenando [guardando] meus itens de higiene menstrual (absorvente/coletor menstrual/tampão/pano de absorção menstrual e/ou outros) limpos ou que sobraram até meu ciclo seguinte*				
***11-*** *Eu pude lavar as mãos quando quis*				
***12-*** *Eu pude descartar imediatamente meus itens de higiene menstrual (absorvente/coletor menstrual/tampão/pano de absorção menstrual e/ou outros) usados*				
***13-*** *Eu pude descartar meus itens de higiene menstrual (absorvente/coletor menstrual/tampão/pano de absorção menstrual e/ou outros) usados do jeito que eu quis*				
***14-*** *Me preocupei em onde descartar os meus itens de higiene menstrual (absorvente/coletor menstrual/tampão/pano de absorção menstrual e/ou outros) usados*				
***15-*** *Me preocupei que outras pessoas pudessem ver meus itens de higiene menstrual (absorvente/coletor menstrual/tampão/pano de absorção menstrual e/ou outros) usados no lugar onde os descartei*				
***Em casa, durante minha última menstruação…*.**				
***16-*** *Em casa, consegui trocar meus itens de higiene menstrual (absorvente/coletor menstrual/tampão/pano de absorção menstrual e/ou outros) quando quis*				
***17-*** *Em casa, fiquei satisfeita com o lugar onde costumava trocar meus itens de higiene menstrual (absorvente/coletor menstrual/tampão/pano de absorção menstrual e/ou outros)*				
***18-*** *Em casa, eu tinha um lugar limpo para trocar meus itens de higiene menstrual (absorvente/coletor menstrual/tampão/pano de absorção menstrual e/ou outros)*				
***19-*** *Em casa, me preocupei que não pudesse trocar meus itens de higiene menstrual (absorvente/coletor menstrual/tampão/pano de absorção menstrual e/ou outros) quando precisasse*				
***20-*** *Em casa, me preocupei que alguém me visse enquanto trocava meus itens de higiene menstrual (absorvente/coletor menstrual/tampão/pano de absorção menstrual e/ou outros)*				
***21-*** *Em casa, me preocupei que alguém me machucasse enquanto trocava meus itens de higiene menstrual (absorvente/coletor menstrual/tampão/pano de absorção menstrual e/ou outros)*				
***22-*** *Em casa, me preocupei que algo me prejudicasse enquanto trocava meus itens de higiene menstrual (absorvente/coletor menstrual/tampão/pano de absorção menstrual e/ou outros) (por exemplo, animais, insetos, estrutura insegura)*				
***Na escola [trabalho/fora de casa], durante minha última menstruação....***				
***23-*** *Na escola, consegui trocar meus itens de higiene menstrual (absorvente/coletor menstrual/tampão/pano de absorção menstrual e/ou outros) quando quis*				
***24-*** *Na escola, fiquei satisfeita com o lugar que eu usei para trocar meus itens de higiene menstrual (absorvente/coletor menstrual/tampão/pano de absorção menstrual e/ou outros)*				
***25-*** *Na escola, eu tinha um lugar limpo para trocar meus itens de higiene menstrual (absorvente/coletor menstrual/tampão/pano de absorção menstrual e/ou outros)*				
***26-*** *Na escola, me preocupei que não pudesse trocar meus itens de higiene menstrual (absorvente/coletor menstrual/tampão/pano de absorção menstrual e/ou outros) quando precisasse*				
***27-*** *Na escola, me preocupei que alguém me visse enquanto trocava meus itens de higiene menstrual (absorvente/coletor menstrual/tampão/pano de absorção menstrual e/ou outros)*				
***28-*** *Na escola, me preocupei que alguém me prejudicasse enquanto trocava meus itens de higiene menstrual (absorvente/coletor menstrual/tampão/pano de absorção menstrual e/ou outros)*				
***Se você lavou e reutilizou qualquer produto durante sua última menstruação, por favor, responda a estes itens.***				
***Durante minha última menstruação…*.**				
***29-*** *Eu tive água suficiente para lavar meu item de higiene menstrual (absorvente/coletor menstrual/tampão/pano de absorção menstrual e/ou outros) ou deixá-lo de molho*				
***30-*** *Eu tive acesso a uma pia para lavar meus itens de higiene menstrual (absorvente/coletor menstrual/tampão/pano de absorção menstrual e/ou outros) ou deixá-los de molho sempre que precisava*				
***31-*** *Eu pude lavar meus itens de higiene menstrual (absorvente/coletor menstrual/tampão/pano de absorção menstrual e/ou outros) quando quis*				
***32-*** *Eu tive sabão suficiente para lavar meus itens de higiene menstrual (absorvente/coletor menstrual/tampão/pano de absorção menstrual e/ou outros)*				
***33-*** *Eu pude secar meus itens de higiene menstrual (absorvente/coletor menstrual/tampão/pano de absorção menstrual e/ou outros) quando eu quis*				
***34-*** *Eu pude secar meus itens de higiene menstrual (absorvente/coletor menstrual/tampão/pano de absorção menstrual e/ou outros) quando eu quis*				
***35-*** *Me preocupei que meus itens de higiene menstrual (absorvente/coletor menstrual/tampão/pano de absorção menstrual e/ou outros) não estivessem secos quando eu precisasse deles*				
***36-*** *Me preocupei que outros vissem meus itens de higiene menstrual (absorvente/coletor menstrual/tampão/pano de absorção menstrual e/ou outros) enquanto secavam*				

In the pre-final stage, the nearly finalized version of the instrument was administered to 360 female high school students from two public schools. Participants’ mean age was 17.18 years (95% CI: 17.02-17.34). The majority were single (91.9%), of mixed race (49.4%), religiously affiliated (79.7%), and living in urban areas (84.4%). Small percentages lived in rural areas (15%) or in indigenous or *quilombola* communities (0.3% each). The age at first menstruation ranged from 11 years old (22.3%) to 12 years old (31.8%). Disposable pads were the most commonly used method during the last menstrual period (70.5%), followed by toilet paper (10.3%), other methods (9.4%), tampons/pads (5%), menstrual panties (3.3%), and menstrual cups (1.5%). The main reasons for absence from daily activities were cramps (59%) and heavy menstrual flow (37.5%).

The instrument was validated using Cronbach’s alpha to assess reliability and internal consistency based on participants’ responses to MPNS-36. The results revealed an overall Cronbach’s alpha value of 0.78 for the scale. There were no suggestions for modifications or doubts about the questions answered by participants, allowing the scale applied to the pre-test population to be maintained without changes. After following all the necessary stages and respecting scientific rigor, the MPNS-36 Brazilian version was submitted to the author of the original instrument, who approved the final version.

## DISCUSSION

This study translated, culturally adapted, and validated the MPNS-36 Brazilian Portuguese version, which is a self-report tool developed to assess the extent to which an individual’s menstrual management practices and environments meet their perceived needs^(7)^. Its translation and cultural adaptation were carefully conducted based on prior research, with special attention to encompass the Brazilian population’s cultural diversity. The finalized tool reflects a comprehensive approach that incorporates diverse experiences regarding menstrual practices. In addition to the original English version, it has been culturally adapted and validated in several languages, including Ateso, the primary language of Uganda and the language used in the original study. It has also been translated into Spanish but has not yet been validated^(14)^.

Poverty or menstrual insecurity affects countless people’s physical and mental health, with the main challenges of menstrual management including cultural barriers, lack of education and stigmatization, as well as practical issues such as inadequate access to products and infrastructure. These factors negatively affect health and participation in educational and professional activities, and symptoms can even cause absenteeism from school and work^(6)^.

The constant gap and neglect of intimate and menstrual hygiene, often considered taboo, highlights the need for a more rigorous research instrument. Unlike the predominantly qualitative or secondary studies carried out in Brazil, an instrument that provides objective and detailed data can offer a broader and more accurate understanding of the menstrual issue, allowing an analysis of the singularities of each person investigated^(4)^.

Menstruation is directly related to human dignity and the United Nations (UN) Sustainable Development Goals (SDGs). The UN lists 17 SDGs. Among them, it is understood that the right to menstrual hygiene is strongly connected to the first goal, which refers to eradication of poverty; the third goal, which refers to achieving health and well-being; the fourth goal, which concerns quality education; the fifth goal, which deals with gender equality; the sixth goal, which refers to access to sanitation and clean water; and the tenth goal, which concerns reduction of inequalities^(15)^.

After undergoing the methodological process of translation, cultural adaptation and validity, recommended in literature^(10)^, the scale presented an overall CVI of 97%, indicating a remarkably high score. This robust score suggests, according to the assessment of the experts involved, that the instrument items are highly relevant and pertinent to the construct being measured. This finding strengthens confidence in the instrument quality, indicating solid content validity^(11,16)^.

When dealing with the instrument’s subscales, individual and total CVI values exceed 90%, suggesting items of extreme importance for assessing the measured constructs. The original study for the creation and validity of MPNS-36 used the Intraclass Correlation Coefficient (ICC), another evidence of validity of content of a test, also reflecting high levels of reliability and test-retest stability. The total ICC of the study’s subscales exceeded 95%, which indicates excellent agreement in the study^(7)^. These results strengthen the credibility and usefulness of the original and adapted MPNS-36 in Brazilian Portuguese as a reliable and valid tool for assessing the proposed constructs.

Cronbach’s alpha coefficient is important for assessing the reliability of measuring instruments^(13)^. The MPNS-36 internal consistency analysis revealed a total coefficient of 0.781. This value, located in the range of 0 to 1, indicates an acceptable internal consistency, with values closer to 1 suggesting greater reliability and cohesion between the instrument’s items. Cronbach’s alpha obtained suggests that the items are positively correlated, reflecting good cohesion. Additionally, the specific value of Cronbach’s alpha, calculated from the 36 standardized items, was 0.784, which reinforces the scale’s robustness and reliability^(13,17)^.

When the subscales of the adapted instrument were analyzed, it showed better results than the original study, with total alpha values above 0.77. The improved reliability of the “transport and school environment needs”, “material and reliability concerns” and “reuse needs” subscales in the adapted instrument stands out, compared to the original scale, in which the indexes were 0.66, 0.51 and 0.47, respectively^(7)^. These results demonstrate more robust evidence of reliability in the crucial areas of the adapted instrument, strengthening its validity and usefulness in the Brazilian context.

In this regard, MPNS-36 - Brazilian version presented evidence of satisfactory internal consistency and acceptable reliability for measuring girls’ and women’s menstrual needs and realities in Brazil.

Furthermore, the MPNS-36 pre-test phase results demonstrated that the instrument adequately captures the nuances of menstrual needs among Brazilian adolescents. The sample, composed of high school students with an average age of 17.18 years, reflected the typical sociodemographic profile of young Brazilian women: mostly single, brown, religious, and living in urban areas. The prevalence of disposable pads as a menstrual management method, followed by alternative practices such as toilet paper use, suggests economic and educational barriers to menstrual product access. Additionally, the low use of menstrual cups and tampons may be related to cultural taboos and a lack of information, underscoring the necessity of educational interventions^(18,19)^.

The identification of the main reasons for absence from daily activities, such as intense menstrual cramps and heavy flow, highlights the importance of addressing the appropriate management of menstruation to improve female adolescents’ quality of life. These results underscore the relevance of a validated and culturally adapted instrument, such as MPNS-36, that can offer an accurate and reliable assessment of menstrual needs^(18)^. The implementation of such a tool could inform public policies and educational programs focused on menstrual health in Brazil, promoting a more inclusive and informed environment that meets adolescents’ real needs in managing menstruation.

### Study limitations

The main limitation of this study was adjusting the questionnaire to account for Brazil’s linguistic, cultural, and regional diversity to ensure comprehension throughout the country. Careful consideration of regional specificities, linguistic variations, and cultural nuances was required to address this challenge. Additionally, the pre-test data was collected in a single municipality and practice setting. While this complies with the purpose of the methodological study, it does not allow for generalization of results.

Therefore, future research should be conducted with other population groups, such as young adult women and indigenous communities, as well as in different regions of the country. This will broaden the scale’s cultural, age, and socioeconomic scope. It is essential that the instrument’s psychometric properties be reassessed in these studies to confirm its robustness and applicability to different population profiles. These efforts will strengthen the evidence of the scale’s validity and promote a more comprehensive understanding of menstrual management practices in Brazil.

### Contributions to nursing, health and public policies

This instrument’s relevance is evident in its ability to deepen understanding of the menstrual needs and realities of the Brazilian population. It generates indicators that can guide the development of public policies addressing menstrual issues. As previously mentioned, the scarcity of quantitative studies in Brazil hinders a thorough analysis of menstrual poverty, which, in turn, impedes the development of effective public policies. This study therefore represents an opportunity to address this issue by using a validated instrument. In clinical nursing practice, the instrument’s validity will enable healthcare professionals to more accurately identify patients’ menstrual needs, facilitating the provision of targeted, effective care. The result will be personalized interventions that respect the particularities of each patient, improving care and promoting a comprehensive approach to menstrual health. Furthermore, by providing concrete data, the instrument will contribute to the implementation of evidence-based practices, thereby reinforcing the quality of care provided in nursing.

## CONCLUSIONS

MPNS-36 - Brazilian version was considered culturally adapted and validated, and can be used in Brazil to analyze the menstrual needs and reality of the Brazilian population.

## Data Availability

The research data are available only upon request.
