# ERRATUM

**DOI:** 10.1590/0034-7167.202679e0120240606

**Published:** 2026-07-10

In the article “Translation, cross-cultural adaptation and validity study of the Menstrual Practice Needs Scale (MPNS-36)”, with DOI number: https://doi.org/10.1590/0034-7167-2024-0606, published in Revista Brasileira de Enfermagem, 2025;78(Suppl 4):e20240606, on page 6, Chart 1:

Where it read:

**34**- Eu pude secar meus itens de higiene menstrual (absorvente/coletor menstrual/tampão/pano de absorção menstrual e/ou outros) quando eu quis

Read:

**34**- Me preocupei que alguém me visse enquanto eu lavava meus itens de higiene menstrual (absorvente/coletor menstrual/tampão/pano de absorção menstrual e/ou outros)

**Editor in Chief** Prof. PhD Dulce Barbosa

