# Supplementary material for: Translation, cross-cultural adaptation and validity study of the Menstrual Practice Needs Scale (MPNS-36)
Source: Rev Bras Enferm. 2025 Dec 8;78(Suppl 4):e20240606. doi: 10.1590/0034-7167-2024-0606 (PMC12700574; doi:10.1590/0034-7167-2024-0606)
Supplement: Translation

# Translation: Tradução, adaptação transcultural e estudo de validação da *Menstrual Practice Needs Scale* (MPNS-36)

**DOI:** 10.1590/0034-7167-2024-0606pt

**Authors:** Maria Eduarda Pascoaloto da Silva, Evelly Vitória Azevedo de Souza, Bianca Dargam Gomes Vieira, Mariana Lourenço Haddad, Ana Paula de Assis Sales, Sonia Silva Marcon, Catchia Hermes-Uliana, Mara Cristina Ribeiro Furlan

**Affiliations:** I Universidade Estadual de Maringá. Maringá, Paraná, Brasil; II Universidade Federal da Grande Dourados. Dourados, Mato Grosso do Sul, Brasil; III Universidade Federal Fluminense. Niterói, Rio de Janeiro, Brasil; IV Universidade Estadual de Londrina. Londrina, Paraná, Brasil; V Universidade Federal de Mato Grosso do Sul. Três Lagoas, Mato Grosso do Sul, Brasil

**Keywords:** Menstruação, Estudos de Validação, Produtos de Higiene Menstrual, Tradução, Saúde da Mulher.

## Abstract

**Objetivos:**

realizar a tradução, adaptação transcultural e validação da *Menstrual Practice Needs Scale* para o contexto brasileiro.

**Métodos:**

estudo metodológico, seguindo as etapas: tradução inicial; síntese; retrotradução; síntese; revisão por comitê de *experts*; pré-teste; e submissão e aprovação pelo autor. O comitê foi composto por 11 juízes. A coleta do pré-teste foi realizada de julho a outubro de 2023 em município de Mato Grosso do Sul, com 360 alunas do ensino médio. A análise utilizou o Índice de Validade de Conteúdo e o alfa de Cronbach.

**Resultados:**

o comitê alcançou um Índice de Validade de Conteúdo de 96,7%, porém ajustes sugeridos pelos juízes foram incorporados sem nova análise. A versão foi aprovada para o pré-teste, apresentando um alfa de Cronbach de 0,78, sem dúvidas ou sugestões de modificação.

**Conclusões:**

a adaptação transcultural e a validação da escala para o português do Brasil foram consideradas adequadas para a aplicação na população-alvo.

## INTRODUÇÃO

A higiene íntima menstrual é crucial para o bem-estar físico, psicológico e espiritual das mulheres, sendo amplamente reconhecida como um direito fundamental. Sua adequada gestão requer a disponibilidade de ambientes e métodos higiênicos apropriados, além de materiais e informações adequadas^(1,2)^. A situação conhecida como “pobreza menstrual” ou “precariedade menstrual” refere-se à vulnerabilidade econômica e social que afeta bilhões de pessoas menstruantes globalmente, devido à falta de acesso a saneamento básico, banheiros e itens essenciais como os absorventes^(3)^.

No Brasil, mais de 713 mil meninas vivem sem banheiros domiciliares, e cerca de 200 mil alunas enfrentam condições precárias para gerenciar a menstruação na escola, o que pode provocar sofrimento emocional e impactar negativamente o desenvolvimento das mulheres^(4)^. Em resposta a essa realidade, a Lei nº 14.214, promulgada em 2021 e regulamentada pelo Decreto nº 11.432 em março de 2023, instituiu a distribuição gratuita de absorventes e cuidados relacionados à saúde menstrual^(5)^. No entanto, a implementação dessas políticas ainda é insuficiente, pois os absorventes representam apenas um dos muitos recursos necessários para garantir a dignidade menstrual^(2,6)^.

Esse cenário está diretamente relacionado à falta de estudos que reflitam adequadamente a situação da pobreza menstrual no Brasil e que possibilitem o desenvolvimento de políticas públicas adequadas à realidade nacional. O relatório “Pobreza Menstrual no Brasil”, elaborado pelo Fundo das Nações Unidas para a Infância e pelo Fundo de População das Nações Unidas, em 2021, apontou as dificuldades enfrentadas para analisar a realidade do país em relação às necessidades menstruais^(4)^. Um dos principais objetivos do relatório foi destacar a urgência de conduzir mais pesquisas quantitativas e realizar uma coleta de dados abrangente para preencher essa lacuna e fornecer uma visão da pobreza menstrual no Brasil.

Dessa forma, após revisão da literatura, identificou-se como lacuna no conhecimento a falta de instrumentos válidos em língua portuguesa para medir as necessidades de higiene menstrual de meninas e mulheres brasileiras. Essa ausência dificulta a correlação dos dados nacionais com resultados de diferentes culturas. No entanto, na busca por instrumentos adequados, levou-se à descoberta da *Menstrual Practice Needs Scale* (MPNS-36) na literatura internacional. Desenvolvida e validada em Soroti, Uganda, esta escala fornece uma base sólida para avaliar as necessidades menstruais, possibilitando a adaptação para o contexto brasileiro^(7)^.

Avaliar os fatores que influenciam os hábitos e práticas de higiene menstrual requer o uso de instrumentos válidos para garantir análises precisas. A coleta de dados com instrumentos validados em português orienta a prática da enfermagem, contribuindo para o desenvolvimento de tecnologias de saúde e a melhoria da qualidade da assistência prestada^(8,9)^. Assim, a escassez de traduções e adaptações transculturais de instrumentos de medida nesta área evidenciam a necessidade desta pesquisa.

## OBJETIVOS

Realizar a tradução, adaptação transcultural e validação da MPNS-36 para o contexto brasileiro.

## MÉTODOS

### Aspectos éticos

Para a condução deste estudo, foi obtida a autorização da autora correspondente da escala para proceder com a tradução, adaptação cultural e validação do instrumento. O estudo foi submetido e aprovado pelo Comitê de Ética em Pesquisa da Universidade Federal de Mato Grosso do Sul, sob Parecer n^o^ 5.652.278, cumprindo com todas as exigências éticas e legais das pesquisas que envolvem seres humanos.

### Desenho, período e local do estudo

Trata-se de estudo metodológico de abordagem quantitativa, cujo referencial metodológico utilizado foi proposto por Beaton *et al*.^(10)^. Para a efetivação do processo, as seguintes etapas foram seguidas: 1) tradução inicial; 2) síntese da tradução; 3) retrotradução; 4) síntese da retrotradução; 5) revisão pelo comitê de *experts* (juízes); 6) realização do pré-teste; 7) submissão e aprovação pelo autor. A coleta de dados para realização da etapa do pré-teste foi realizada de julho a outubro de 2023 em duas escolas estaduais de ensino médio em município localizado na mesorregião do leste do estado do Mato Grosso do Sul. A elaboração e condução deste estudo seguiram as diretrizes do *COnsensus-based Standards for the selection of health Measurement INstruments*, com o objetivo de garantir a qualidade metodológica nas etapas de tradução, adaptação cultural e avaliação das propriedades psicométricas do instrumento.

### População ou amostra; critérios de inclusão e exclusão

A população do estudo foi composta por quatro tradutores, sendo dois responsáveis pela primeira etapa de tradução e dois pela retrotradução, além de 11 juízes avaliadores. Embora não haja consenso na literatura sobre o número exato de juízes a serem incluídos no comitê de *experts*, é fundamental que os membros sejam especialistas na área do estudo e fluentes no idioma original do instrumento^(7)^. Para a análise pelo comitê, a seleção dos juízes foi intencional e realizada por amostragem não probabilística por conveniência, levando em consideração a relevância das qualificações e a especialização dos profissionais na temática. O comitê foi composto por um tradutor de cada fase da tradução, um profissional da área da linguística, um metodologista e especialistas da área da saúde/enfermagem^(8,11)^.

A amostra do pré-teste incluiu 360 participantes, distribuídas entre duas escolas estaduais de ensino médio: 86 alunas da escola A (35 do 1º ano, 24 do 2º ano e 27 do 3º ano) e 274 alunas da escola B (88 do 1º ano, 88 do 2º ano e 98 do 3º ano). Optou-se por definir a amostra do pré-teste através da proporção de 10:1, conforme recomendado pelo estudo da escala original^(7)^. Foram incluídas adolescentes do sexo feminino, matriculadas no ensino médio das escolas selecionadas, que já haviam passado pela menarca. Não foram incluídas aquelas cuja menarca havia ocorrido há menos de seis meses ou que apresentavam dificuldades cognitivas que pudessem comprometer a compreensão e o preenchimento do instrumento.

### Instrumento de coleta de dados

A MPNS-36 foi desenvolvida e validada em Soroti, Uganda^(7)^. Ela pode ser aplicada de forma autoaplicável ou através de entrevista. A escala utiliza um formato tipo Likert de três pontos, com as respostas como “nunca” (0), “às vezes” (1), “frequentemente” (2) e “sempre” (3). A MPNS-36 é composta por seis dimensões, ou subescalas, que agrupam itens para capturar informações específicas sobre as práticas menstruais. Entre essas subescalas, quatro são aplicáveis a todas as entrevistadas, enquanto duas são relevantes apenas para aquelas que lavam ou reutilizam seu material menstrual^(7)^.

As subescalas abrangem tópicos como ambiente doméstico, transporte e ambiente escolar, confiabilidade do material, insegurança com mudanças e descarte, e necessidades e inseguranças na reutilização do material^(7)^. A pontuação da escala é calculada pela média das respostas dos itens relevantes. As opções de resposta têm valores específicos, sendo “nunca“ equivalente a 0, “às vezes“, a 1, “frequentemente“, a 2, e “sempre“, a 3. Pontuações mais elevadas indicam uma experiência menstrual mais positiva, enquanto uma pontuação de 3 sugere que o entrevistado não apresenta necessidades não atendidas e tem uma realidade menstrual bem atendida^(7)^.

### Protocolo do estudo

A primeira etapa envolveu a tradução do instrumento para o português do Brasil, realizada por dois tradutores bilíngues (T1 e T2). Os critérios de inclusão para os tradutores eram ter o português como idioma materno e possuir proficiência em inglês. A T1 era uma profissional brasileira, da área da enfermagem, com mestrado e doutorado na área da saúde da mulher, com suficiência na língua inglesa e experiência em processos de tradução e adaptação cultural. O T2 foi uma profissional juramentada, de naturalidade brasileira, com graduação em letras (habilitação em português/inglês), bilíngue nos idiomas português e inglês, e experiência na tradução, versão e revisão de artigos científicos, livros e documentos em geral. Essa escolha visou minimizar o risco de vieses linguísticos, psicológicos, culturais e de compreensão teórica e prática. Com a versão do tradutor 1 (T1) e a versão do tradutor 2 (T2) em mãos, foram realizadas análises de discrepâncias entre as versões pelo pesquisador e tradutores. Após a análise, deu-se origem à síntese dessas versões, denominada versão T1-2. Essa comparação visou integrar as duas traduções e resolver quaisquer discrepâncias, resultando em uma versão única.

Posteriormente, fez-se a retrotradução (RT) ou *back-translation* da versão T1-2, em que dois tradutores (RT1 e RT2) bilíngues, com o idioma de origem da escala como sua língua materna (inglês) e proficiência na língua portuguesa, realizaram a tradução reversa (português do Brasil → inglês). Ambas as retrotradutoras possuíam nacionalidades brasileira e americana, e eram falantes nativa de português e inglês. Já na etapa de síntese de retrotradução, os tradutores, juntamente com os responsáveis pela pesquisa, realizaram a comparação dos instrumentos, para verificar as semelhanças, não necessariamente literárias, mas para perceber se existia concordância entre as versões, e, em caso de discrepâncias, realizaram a correção em consenso, com o objetivo de garantir que a versão traduzida refletisse o mesmo conteúdo da versão original. Após a análise, deu-se origem à síntese dessas versões, denominada versão RT1-2.

A terceira etapa consistiu na avaliação das equivalências semântica, idiomática, cultural e conceitual da MPNS-36. Essa avaliação foi realizada por um comitê de especialistas em duas fases: a primeira envolveu a revisão de todo o material produzido por meio de um formulário eletrônico; a segunda foi uma reunião entre as pesquisadoras para resolver discrepâncias e obter consenso sobre a versão final.

O comitê de especialistas foi composto por 11 profissionais da área da saúde e/ou docentes de enfermagem, com pelo menos três anos de experiência em saúde, saúde pública ou saúde da mulher, além de experiência com tradução e adaptação cultural, e um linguista. A experiência com tradução e adaptação cultural foi definida com base na participação anterior dos especialistas em estudos metodológicos sobre o tema e/ou publicações relacionadas, desde que tivessem contato prévio com esse tipo de processo, sem a exigência de tempo mínimo ou quantidade específica de produções. A seleção desses profissionais foi feita através da Plataforma *Lattes* e de indicações^(12)^.

Os juízes avaliaram o instrumento com uma escala Likert de 1 a 4, focando em quatro tipos de equivalência: semântica (preservação do significado das palavras); idiomática (adequação de expressões e coloquialismos ao idioma-alvo); cultural (adaptação de termos e situações para o contexto cultural); e conceitual (representação correta de conceitos culturais específicos)^(8)^.

O formulário de avaliação foi disponibilizado no *Google Forms^®^*, organizado em capítulos e subdividido em parágrafos, para facilitar a análise. Os especialistas tiveram 30 dias para completar a avaliação e foram solicitados a fornecer comentários sempre que a pontuação atribuída fosse inferior a 4. A análise dos itens seguia uma escala de quatro níveis de concordância: 1 = não equivalente ou não claro; 2 = necessita de grande revisão ou é pouco claro; 3 = equivalente, necessita de pequenas alterações ou é bastante claro; 4 = absolutamente equivalente ou muito claro^(8,11)^. O formulário também incluía um espaço para sugestões específicas. Após a coleta das avaliações, as sugestões foram analisadas, e apenas os itens com concordância mínima de 80% foram aceitos. Itens que não atingiram essa porcentagem foram reavaliados pelo comitê de *experts* até alcançarem a concordância requerida.

A última etapa do estudo envolveu a aplicação da versão pré-final da escala a 360 alunas do ensino médio de duas escolas selecionadas. A equipe responsável foi treinada para garantir a correta utilização do instrumento. A aplicação foi autorizada pelo comitê de ética e pela Secretaria de Estado de Educação de Mato Grosso do Sul, após acordos com diretores, coordenadores e discussões com professores das turmas envolvidas. Os alunos foram informados sobre o objetivo, desenvolvimento, riscos e benefícios da pesquisa. Reuniões foram agendadas para fornecer informações adicionais e esclarecer dúvidas. Durante essas reuniões, os termos de assentimento e consentimento foram disponibilizados, concedendo um prazo mínimo de uma semana, para que os pais ou responsáveis assinassem e entregassem os documentos à equipe de pesquisa. A coleta de dados começou apenas após a conclusão dessas etapas.

Os dados foram coletados utilizando *tablets* e celulares das participantes conectados à *internet* via código QR fornecido pelos aplicadores. O formulário, disponibilizado *online* na plataforma *Google Forms* ^®^, apresentou a escala original e incluiu um espaço para sugestões ou dúvidas após cada questão. Ao final, os aplicadores confirmavam o preenchimento e informavam os participantes que os resultados seriam enviados por e-mail, incentivando-os a entrar em contato com a equipe para esclarecimentos adicionais. A coleta de dados ocorreu de julho a outubro de 2023, e todos os itens do formulário eram obrigatórios, exceto os comentários e questões de reutilização dos materiais. Os procedimentos para tradução, adaptação cultural e validação foram descritos na figura abaixo (Figura 1).

**Figura 1:** Processo de tradução, adaptação cultural e validação da Escala de necessidades e realidade menstrual (MPNS-36) - versão brasileira

*T1 - tradução 1; T2 - tradução 2; RT1 - retrotradução 1; RT2 - retrotradução 2.*

### Análise dos resultados e estatística

Os dados coletados foram organizados em uma planilha no *Microsoft Excel* ^®^ e exportados para o *Statistical Package for the Social Sciences*, versão 25.0.0.0, para análise estatística. A validade do conteúdo foi avaliada usando o Índice de Validade de Conteúdo (IVC), calculado pela proporção de itens com pontuação “3“ ou “4“ na escala de clareza. Itens com pontuação abaixo de “4“ foram revisados com base nas sugestões dos juízes. Um índice de concordância aceitável foi estabelecido em no mínimo 0,80^(8,10,11)^. A confiabilidade e consistência interna dos itens da escala foram analisadas para as respostas da população do pré-teste através do alfa de Cronbach, que varia de 0 a 1, onde valores próximos a 1 indicam maior consistência interna^(13)^.

## RESULTADOS

A MPNS-36 foi adaptada e validada para a cultura brasileira, recebendo o título de “Escala de Necessidades e Realidade Menstrual (MPNS-36) - Versão Brasileira”. A versão brasileira está estruturada em seis dimensões ou subescalas: material e ambiente doméstico; transporte e ambiente escolar; preocupações com a confiabilidade do material; insegurança com mudanças e descarte; necessidades de reutilização do material; e insegurança na reutilização do material.

Na primeira etapa, as traduções realizadas por T1 e T2 foram sintetizadas na versão T12. Ajustes foram necessários para resolver discrepâncias semânticas relacionadas aos termos “itens de higiene menstrual”, “período” e “ciclo menstrual”, alinhando-os com a linguagem da escala original, dando origem à T1-2. A retrotradução gerou RT1 e RT2. Ambas as retrotraduções mostraram alta semelhança com a versão original, mas pequenas discrepâncias foram identificadas. A versão final foi escolhida com base na clareza e na ausência de abreviações; por exemplo, onde a RT1 utilizou “*e.g*.”, que é uma abreviação para “por exemplo”, optou-se pela versão da RT2, onde a mesma utilizou “*for example*”. Questões que se adequavam melhor a tempos verbais e linguagem mais claras foram utilizadas, chegando, assim, a uma versão síntese das retrotraduções (RT1-2).

A avaliação das equivalências semântica, idiomática, cultural e conceitual da MPNS-36 foi conduzida por um comitê de 11 especialistas. Entre eles, sete (63,6%) eram enfermeiras e quatro (36,4%) eram profissionais de letras, todos com proficiência em inglês. Três (27,3%) tinham experiência em estudos de tradução e adaptação cultural, e quatro (36,4%) eram enfermeiras especializadas em saúde da mulher. Quanto à titulação, uma possuía graduação (9,1%), três eram especialistas (27,3%), três eram mestres (27,3%) e quatro eram doutoras (36,4%), com idades entre 28 e 50 anos.

A avaliação resultou em um IVC geral de 96,7%. Os IVCs específicos foram semântica (96,5%), idiomática (94,9%), cultural (96,7%) e conceitual (98,5%). Apesar dos índices superiores a 80%, os juízes especialistas fizeram considerações importantes, levando a ajustes significativos na versão traduzida e adaptada da escala para o português, porém não foi necessária uma nova análise. As observações dos juízes incluíram correções gramaticais, substituições de termos coloquiais e sugestões regionais. O título foi uma das áreas mais modificadas, com duas sugestões que aprimoraram a compreensão do que a escala pretende avaliar, resultando na troca de “prática” por “realidade” menstrual.

Além disso, foi observada a inadequação do termo “itens de higiene menstrual” no contexto brasileiro, o que levou à inclusão de uma explicação detalhada dentro da escala, mencionando exemplos como absorventes e coletores menstruais, ficando, então, desta forma: absorvente/coletor menstrual/tampão/pano de absorção menstrual e/ou outros. A questão 21 teve a palavra “machucasse” substituída por “prejudicasse”, para evitar ambiguidades. Também se decidiu por modificar “basin” para “pia/bacia/tanque”, conforme sugestão dos especialistas. A terminologia “ciclo menstrual” e as opções de resposta “nunca”, “às vezes”, “frequentemente” e “sempre” foram aceitas sem alterações. Foram feitas correções gramaticais e de concordância para melhorar a clareza da escala. Após a revisão, a validade de face da versão adaptada foi confirmada com um IVC total de 97%, indicando um alto nível de concordância entre os juízes^(4,11)^. Assim, alcançou-se consenso sobre a versão final traduzida da MPNS-36 para o português (Quadro 1).

**Quadro 1:** Versão final traduzida da Escala de Necessidades e Realidade Menstrual (MPNS-36) - Versão Brasileira aprovada pelo comitê de *experts*

Escala de Necessidades e Realidade Menstrual MPNS-36 - Versão Brasileira				
Opção de resposta	Nunca	Às vezes	Frequentemente	Sempre
**Durante minha última menstruação...**				
**1-** Meus itens de higiene menstrual (absorvente/coletor menstrual/tampão/pano de absorção menstrual e/ou outros) foram confortáveis				
**2-** Eu tive itens de higiene menstrual (absorvente/coletor menstrual/tampão/pano de absorção menstrual e/ou outros) suficientes para trocar com a frequência que quis				
**3-** Eu fiquei satisfeita com a limpeza dos meus itens de higiene menstrual (absorvente/coletor menstrual/tampão/pano de absorção menstrual e/ou outros)				
**4-** Eu pude obter mais unidades do meu item de higiene menstrual (absorvente/coletor menstrual/tampão/pano de absorção menstrual e/ou outros) quando precisei				
**5-** Me preocupei que meus itens de higiene menstrual (absorvente/coletor menstrual/tampão/pano de absorção menstrual e/ou outros) permitissem que o sangue atravessasse para a parte externa das minhas roupas				
**6-** Me preocupei que meus itens de higiene menstrual (absorvente/coletor menstrual/tampão/pano de absorção menstrual e/ou outros) saíssem do lugar enquanto eu os utilizava				
**7-** Me preocupei em como obter mais itens de higiene menstrual (absorvente/coletor menstrual/tampão/pano de absorção menstrual e/ou outros) se eles acabassem				
**8-** Me senti confortável ao levar meus itens de higiene menstrual (absorvente/coletor menstrual/tampão/pano de absorção menstrual e/ou outros) extras comigo enquanto estava fora de casa				
**9-** Me senti confortável ao levar meus itens de higiene menstrual (absorvente/coletor menstrual/tampão/pano de absorção menstrual e/ou outros) extras comigo até o lugar onde os trocava				
**10-** Me senti confortável armazenando [guardando] meus itens de higiene menstrual (absorvente/coletor menstrual/tampão/pano de absorção menstrual e/ou outros) limpos ou que sobraram até meu ciclo seguinte				
**11-** Eu pude lavar as mãos quando quis				
**12-** Eu pude descartar imediatamente meus itens de higiene menstrual (absorvente/coletor menstrual/tampão/pano de absorção menstrual e/ou outros) usados				
**13-** Eu pude descartar meus itens de higiene menstrual (absorvente/coletor menstrual/tampão/pano de absorção menstrual e/ou outros) usados do jeito que eu quis				
**14-** Me preocupei em onde descartar os meus itens de higiene menstrual (absorvente/coletor menstrual/tampão/pano de absorção menstrual e/ou outros) usados				
**15-** Me preocupei que outras pessoas pudessem ver meus itens de higiene menstrual (absorvente/coletor menstrual/tampão/pano de absorção menstrual e/ou outros) usados no lugar onde os descartei				
**Em casa, durante minha última menstruação…**.				
**16-** Em casa, consegui trocar meus itens de higiene menstrual (absorvente/coletor menstrual/tampão/pano de absorção menstrual e/ou outros) quando quis				
**17-** Em casa, fiquei satisfeita com o lugar onde costumava trocar meus itens de higiene menstrual (absorvente/coletor menstrual/tampão/pano de absorção menstrual e/ou outros)				
**18-** Em casa, eu tinha um lugar limpo para trocar meus itens de higiene menstrual (absorvente/coletor menstrual/tampão/pano de absorção menstrual e/ou outros)				
**19-** Em casa, me preocupei que não pudesse trocar meus itens de higiene menstrual (absorvente/coletor menstrual/tampão/pano de absorção menstrual e/ou outros) quando precisasse				
**20-** Em casa, me preocupei que alguém me visse enquanto trocava meus itens de higiene menstrual (absorvente/coletor menstrual/tampão/pano de absorção menstrual e/ou outros)				
**21-** Em casa, me preocupei que alguém me machucasse enquanto trocava meus itens de higiene menstrual (absorvente/coletor menstrual/tampão/pano de absorção menstrual e/ou outros)				
**22-** Em casa, me preocupei que algo me prejudicasse enquanto trocava meus itens de higiene menstrual (absorvente/coletor menstrual/tampão/pano de absorção menstrual e/ou outros) (por exemplo, animais, insetos, estrutura insegura)				
**Na escola [trabalho/fora de casa], durante minha última menstruação....**				
**23-** Na escola, consegui trocar meus itens de higiene menstrual (absorvente/coletor menstrual/tampão/pano de absorção menstrual e/ou outros) quando quis				
**24-** Na escola, fiquei satisfeita com o lugar que eu usei para trocar meus itens de higiene menstrual (absorvente/coletor menstrual/tampão/pano de absorção menstrual e/ou outros)				
**25-** Na escola, eu tinha um lugar limpo para trocar meus itens de higiene menstrual (absorvente/coletor menstrual/tampão/pano de absorção menstrual e/ou outros)				
**26-** Na escola, me preocupei que não pudesse trocar meus itens de higiene menstrual (absorvente/coletor menstrual/tampão/pano de absorção menstrual e/ou outros) quando precisasse				
**27-** Na escola, me preocupei que alguém me visse enquanto trocava meus itens de higiene menstrual (absorvente/coletor menstrual/tampão/pano de absorção menstrual e/ou outros)				
**28-** Na escola, me preocupei que alguém me prejudicasse enquanto trocava meus itens de higiene menstrual (absorvente/coletor menstrual/tampão/pano de absorção menstrual e/ou outros)				
**Se você lavou e reutilizou qualquer produto durante sua última menstruação, por favor, responda a estes itens.**				
**Durante minha última menstruação…**.				
**29-** Eu tive água suficiente para lavar meu item de higiene menstrual (absorvente/coletor menstrual/tampão/pano de absorção menstrual e/ou outros) ou deixá-lo de molho				
**30-** Eu tive acesso a uma pia para lavar meus itens de higiene menstrual (absorvente/coletor menstrual/tampão/pano de absorção menstrual e/ou outros) ou deixá-los de molho sempre que precisava				
**31-** Eu pude lavar meus itens de higiene menstrual (absorvente/coletor menstrual/tampão/pano de absorção menstrual e/ou outros) quando quis				
**32-** Eu tive sabão suficiente para lavar meus itens de higiene menstrual (absorvente/coletor menstrual/tampão/pano de absorção menstrual e/ou outros)				
**33-** Eu pude secar meus itens de higiene menstrual (absorvente/coletor menstrual/tampão/pano de absorção menstrual e/ou outros) quando eu quis				
**34-** Eu pude secar meus itens de higiene menstrual (absorvente/coletor menstrual/tampão/pano de absorção menstrual e/ou outros) quando eu quis				
**35-** Me preocupei que meus itens de higiene menstrual (absorvente/coletor menstrual/tampão/pano de absorção menstrual e/ou outros) não estivessem secos quando eu precisasse deles				
**36-** Me preocupei que outros vissem meus itens de higiene menstrual (absorvente/coletor menstrual/tampão/pano de absorção menstrual e/ou outros) enquanto secavam				

Na etapa pré-final, a versão quase finalizada do instrumento foi aplicada a 360 alunas do ensino médio de duas escolas da rede estadual. A média de idade das participantes foi de 17,18 anos (IC-95%: 17,02-17,34), sendo a maioria solteira (91,9%), parda (49,4%), pertencente a alguma religião (79,7%) e residente na área urbana (84,4%), com pequenas porcentagens em áreas rurais (15%) e em comunidades indígenas ou quilombolas (0,3% cada). A idade do primeiro sangramento menstrual variou entre 11 (22,3%) e 12 anos (31,8%). O absorvente descartável foi o método mais utilizado no último período menstrual (70,5%), seguido de papel higiênico (10,3%), outros métodos (9,4%), absorvente interno/tampão (5%), calcinha absorvente menstrual (3,3%) e coletor menstrual (1,5%). As principais razões para ausência nas atividades diárias foram cólicas (59%) e alto fluxo menstrual (37,5%).

A validação do instrumento foi realizada utilizando o alfa de Cronbach, para avaliar a confiabilidade e consistência interna com base nas respostas das participantes à MPNS-36. Os resultados revelaram um valor global do alfa de Cronbach de 0,78 para a escala. Não houve sugestões de modificação ou dúvidas nas questões respondidas pelas participantes, permitindo que a escala aplicada na população do pré-teste fosse mantida sem alterações. Após seguir todas as etapas necessárias e respeitar o rigor científico, a versão brasileira da MPNS-36 foi submetida ao autor do instrumento original, que aprovou a versão final.

## DISCUSSÃO

Este estudo traduziu, adaptou culturalmente e validou a versão em português do Brasil da MPNS-36, que é uma ferramenta de autorrelato desenvolvida para avaliar em que medida as práticas e ambientes de gestão menstrual de um indivíduo atendem às suas necessidades percebidas^(7)^. Sua tradução e adaptação cultural foram cuidadosamente conduzidas com base em pesquisa prévia, com especial atenção para abranger a diversidade cultural da população brasileira. A finalização da ferramenta reflete uma abordagem abrangente que incorpora experiências diversas em relação às práticas menstruais. Essa, além do idioma original em inglês, foi adaptada culturalmente e validada em alguns idiomas, como o ateso, língua predominante da Uganda e utilizada no estudo original, traduzida e adaptada para o espanhol, ainda sem validação^(14)^.

A pobreza ou precariedade menstrual afeta a saúde física e psíquica de inúmeras pessoas, sendo que os principais desafios da gestão menstrual incluem barreiras culturais, falta de educação e estigmatização, além de questões práticas como acesso inadequado a produtos e infraestrutura. Esses fatores afetam negativamente a saúde e participação em atividades educacionais e profissionais, podendo os sintomas, inclusive, causar absenteísmo escolar e laboral^(6)^.

A constante lacuna e o esquecimento sobre a higiene íntima e menstrual, frequentemente considerados tabus, destacam a necessidade de um instrumento de investigação mais rigoroso. Ao contrário dos estudos predominantemente qualitativos ou secundários realizados no Brasil, um instrumento que forneça dados objetivos e detalhados pode oferecer uma compreensão mais ampla e precisa da questão menstrual, permitindo uma análise das singularidades de cada pessoa investigada^(4)^.

A passagem pelo período menstrual está diretamente relacionada à dignidade humana e aos Objetivos de Desenvolvimento Sustentável (ODS) da Organização das Nações Unidas (ONU). São 17 os ODS elencados pela ONU. Entre eles, entende-se que o direito à higiene menstrual está fortemente conectado ao primeiro objetivo, referente à erradicação da pobreza; ao terceiro objetivo, referente ao objetivo de alcançar saúde e bem-estar; ao quarto objetivo, concernente à educação de qualidade; ao quinto objetivo, que trata da igualdade de gênero; ao sexto objetivo, referente ao acesso à saneamento e água tratada; e ao décimo objetivo, em relação à redução das desigualdades^(15)^.

Ao passar pelo processo metodológico de tradução, adaptação cultural e validação, preconizado na literatura^(10)^, a escala apresentou um IVC global de 97%, indicando uma pontuação notavelmente elevada. Essa pontuação robusta sugere, conforme avaliação dos especialistas envolvidos, que os itens do instrumento são altamente relevantes e pertinentes ao construto que está sendo medido. Essa constatação fortalece a confiança na qualidade do instrumento, indicando uma sólida validade de conteúdo^(11,16)^.

Ao se tratar das subescalas do instrumento, observam-se valores de IVC individual e total que ultrapassam os 90%, sugerindo itens de extrema importância para avaliação dos construtos medidos. O estudo original de criação e validação da MPNS-36 trabalha com o Coeficiente de Correlação Intraclasse (ICC), outra evidência de validação do conteúdo de um teste, refletindo também em altos índices de confiabilidade e estabilidade teste-reteste. O ICC total das subescalas do estudo superou os 95%, que denota uma concordância excelente do estudo^(7)^. Esses resultados fortalecem a credibilidade e a utilidade da MPNS-36 original e adaptada em português como ferramenta confiável e válida para avaliação dos construtos propostos.

O coeficiente alfa de Cronbach é importante para avaliar a confiabilidade dos instrumentos de medida^(13)^. A análise da consistência interna da MPNS-36 revelou um coeficiente total de 0,781. Este valor, situado no intervalo de 0 a 1, indica uma consistência interna aceitável, com valores mais próximos de 1 sugerindo maior confiabilidade e coesão entre os itens do instrumento. O alfa de Cronbach obtido sugere que os itens estão positivamente correlacionados, refletindo uma boa coesão. Adicionalmente, o valor específico do alfa de Cronbach, calculado a partir dos 36 itens padronizados, foi de 0,784, o que reforça a robustez e confiabilidade da escala^(13,17)^.

Quando analisadas as subescalas do instrumento adaptado, este apresentou melhores resultados diante do estudo original, com valores totais de alfa acima de 0,77. Destaca-se a confiabilidade aprimorada nas subescalas “transporte e ambiente escolar”, “preocupações com confiabilidade do material” e “insegurança na reutilização” no instrumento adaptado, em comparação com a escala original, em que os índices eram de 0,66, 0,51 e 0,47, respectivamente^(7)^. Esses resultados demonstram evidências de confiabilidade mais robustas nas áreas cruciais do instrumento adaptado, fortalecendo sua validade e utilidade no contexto brasileiro.

Nesse sentindo, a MPNS-36 - versão brasileira apresentou evidências de uma consistência interna satisfatória e uma confiabilidade aceitável para mensurar as necessidades e realidades menstruais de meninas e mulheres no Brasil.

Ainda, os resultados da fase pré-teste da MPNS-36 demonstraram que a versão do instrumento é adequada para capturar as nuances das necessidades menstruais das adolescentes brasileiras. A amostra, composta por alunas do ensino médio com média de idade de 17,18 anos, refletiu um perfil sociodemográfico típico de jovens brasileiras, majoritariamente solteiras, pardas, religiosas e residentes em áreas urbanas. A predominância do uso de absorventes descartáveis como método de gestão menstrual, seguido por práticas alternativas como o uso de papel higiênico e outros métodos, sugere a existência de barreiras econômicas e educacionais no acesso a produtos menstruais. Além disso, a baixa utilização de métodos como coletores menstruais e absorventes internos pode estar relacionada a tabus culturais e falta de informação, evidenciando a necessidade de intervenções educativas^(18,19)^.

A identificação das principais razões para ausência nas atividades diárias, como cólicas menstruais intensas e fluxo elevado, destaca a importância de se abordar o manejo adequado da menstruação para melhorar a qualidade de vida dessas adolescentes. Esses resultados sublinham a relevância de um instrumento validado e culturalmente adaptado, como a MPNS-36, que possa oferecer uma avaliação precisa e confiável das necessidades menstruais^(18)^. A implementação de tal ferramenta poderá informar políticas públicas e programas educacionais voltados para a saúde menstrual no Brasil, promovendo um ambiente mais inclusivo e informado que atenda às reais necessidades das adolescentes no manejo da menstruação.

### Limitações do estudo

Este estudo teve como principal limitação a complexidade em ajustar o formulário para a diversidade linguística, cultural e regional do Brasil, a fim de garantir compreensão em todo o território nacional. Este desafio exigiu uma cuidadosa consideração das especificidades regionais, variações linguísticas e nuances culturais presentes em diferentes partes do país. Ademais, a coleta de dados do pré-teste foi realizada em um único município e cenário de prática, o que cumpre com a finalidade do estudo metodológico, entretanto não permite a generalização dos resultados.

Destarte, recomenda-se que futuras pesquisas sejam conduzidas com outros grupos populacionais, como mulheres jovem-adultas e comunidades indígenas, bem como em diferentes regiões do país, a fim de ampliar a abrangência cultural, etária e socioeconômica da escala. Nesses estudos, é fundamental que sejam avaliadas novamente as propriedades psicométricas do instrumento, de modo a confirmar sua robustez e aplicabilidade em distintos perfis populacionais. Esses esforços contribuirão para o fortalecimento das evidências de validade da escala e para uma compreensão mais abrangente das práticas de gestão menstrual no Brasil.

### Contribuições para as áreas da enfermagem, saúde e políticas públicas

A relevância desse instrumento é evidenciada pela sua capacidade de aprofundar a compreensão das necessidades e realidades menstruais da população brasileira, gerando indicadores que podem orientar o desenvolvimento de políticas públicas específicas para as questões menstruais. Conforme discutido, a escassez de estudos quantitativos no Brasil limita a análise abrangente da pobreza menstrual, o que, por sua vez, compromete a criação de políticas públicas eficazes. Este estudo, portanto, representa uma oportunidade para suprir essa lacuna, utilizando um instrumento validado. Na prática clínica de enfermagem, a validação do instrumento permitirá que os profissionais de saúde identifiquem com maior precisão as necessidades menstruais das pacientes, facilitando a oferta de cuidados mais direcionados e eficazes. Isso resultará em intervenções personalizadas que respeitem as particularidades de cada paciente, aprimorando o atendimento e promovendo uma abordagem integral da saúde menstrual. Adicionalmente, ao fornecer dados concretos, o instrumento contribuirá para a implementação de práticas baseadas em evidências, reforçando a qualidade do cuidado prestado na enfermagem.

## CONCLUSÕES

A MPNS-36 - versão brasileira foi considerada adaptada culturalmente e validada, podendo ser utilizada no Brasil para analisar as necessidades e realidade menstruais da população brasileira.

## Data Availability

Os dados de pesquisa só estão disponíveis mediante solicitação.
