# Supplementary material for: ERRATUM
Source: Rev Bras Enferm. 2026 Jul 10;79:e2026e0120240606. doi: 10.1590/0034-7167.202679e0120240606 (PMC13363297; doi:10.1590/0034-7167.202679e0120240606)
Supplement: Related texts

# Correction: ERRATA

**DOI:** 10.1590/0034-7167.202679e0120240606pt

No artigo “Tradução, adaptação transcultural e estudo de validação da Menstrual Practice Needs Scale (MPNS-36)”, com número DOI: https://doi.org/10.1590/0034-7167-2024-0606pt, publicado no periódico Revista Brasileira de Enfermagem, 2025;78(Suppl 4):e20240606, na página, Quadro 1:

Onde se lia:

**34**- Eu pude secar meus itens de higiene menstrual (absorvente/coletor menstrual/tampão/pano de absorção menstrual e/ou outros) quando eu quis

Lê-se:

**34**- Me preocupei que alguém me visse enquanto eu lavava meus itens de higiene menstrual (absorvente/coletor menstrual/tampão/pano de absorção menstrual e/ou outros)

**Editora-chefe** Profa. Dra. Dulce Barbosa
